# CCL21/CCR7 Prevents Apoptosis via the ERK Pathway in Human Non-Small Cell Lung Cancer Cells

**DOI:** 10.1371/journal.pone.0033262

**Published:** 2012-03-16

**Authors:** Ying Xu, Lifeng Liu, Xueshan Qiu, Zihui Liu, Haiying Li, Zixuan Li, Wenting Luo, Enhua Wang

**Affiliations:** 1 Department of Pathology, the First Affiliated Hospital and College of Basic Medical Sciences, China Medical University, Shenyang, Liaoning, China; 2 Institute of Pathology and Pathophysiology, China Medical University, Shenyang, Liaoning, China; 3 Department of Orthopaedics, the First Affiliated Hospital of China Medical University, Shenyang, Liaoning, China; Massachusetts Eye & Ear Infirmary, Harvard Medical School, United States of America

## Abstract

Previously, we confirmed that C-C chemokine receptor 7 (CCR7) promotes cell proliferation via the extracellular signal-regulated kinase (ERK) pathway, but its role in apoptosis of non-small cell lung cancer (NSCLC) cell lines remains unknown. A549 and H460 cells of NSCLC were used to examine the effect of CCL21/CCR7 on apoptosis using flow cytometry. The results showed that activation of CCR7 by its specific ligand, exogenous chemokine ligand 21 (CCL21), was associated with a significant decline in the percent of apoptosis. Western blot and real-time PCR assays indicated that activation of CCR7 significantly caused upregulation of anti-apoptotic bcl-2 and downregulation of pro-apoptotic bax and caspase-3, but not p53, at both protein and mRNA levels. CCR7 small interfering RNA significantly attenuated these effects of exogenous CCL21. Besides, PD98059, a selective inhibitor of MEK that disrupts the activation of downstream ERK, significantly abolished these effects of CCL21/CCR7. Coimmunoprecipitation further confirmed that there was an interaction between p-ERK and bcl-2, bax, or caspase-3, particularly in the presence of CCL21. These results strongly suggest that CCL21/CCR7 prevents apoptosis by upregulating the expression of bcl-2 and by downregulating the expression of bax and caspase-3 potentially via the ERK pathway in A549 and H460 cells of NSCLC.

## Introduction

Chemokines, a family of low-molecular weight pro-inflammatory cytokines, have been described as important mediators not only in cellular trafficking, organ development, tissue remodeling, and tumor metastasis [1]–[3], but in other biological events, such as angiogenesis, lymphopoiesis, proliferations, and apoptosis [4], [5]. Their biological activities are mediated by interaction with the chemokine receptor family of G-protein-coupled receptors (GPCRs) (i.e. C, CC, CXC, and CX3C receptors) [6]. C-C chemokine receptor 7 (CCR7), one of CC chemokine receptors, is expressed on all naive T-cells, some memory T-cells, B-cells, and mature dendritic cells [7]. CCR7 interacted with its ligands, chemokine ligand 19 (CCL19) and chemokine ligand 21 (CCL21) [8], plays important roles in lymphocyte trafficking and homing to lymph nodes during immune and inflammatory reactions [9]–[11].

Our previous study demonstrates that CCR7 is highly expressed in human non-small cell lung cancer (NSCLC) cells, and that CCL21/CCR7 promotes proliferation of A549 and H460 cells of NSCLC via extracellular signal-regulated kinase (ERK) pathway [12], [13]. ERK, a mitogen-activated protein kinases (MAPK) family member, is related to cell proliferation, differentiation, cell cycle regulation, cell survival, and apoptosis [14]–[17]. However, the role of CCR7 in apoptosis of human NSCLC cells has not been elucidated.

The purpose of this study was to examine the effect and regulatory mechanism of the CCL21/CCR7 interaction on apoptosis of A549 and H460 human NSCLC cells. We demonstrated here that CCL21/CCR7 prevents cell apoptosis by upregulating the expression of anti-apoptotic bcl-2 and by downregulating the expression of pro-apoptotic bax and caspase-3, but not p53, which is potentially mediated via the ERK pathway in the NSCLC cells. This study provided novel evidence for the mechanisms of survival of CCR7-mediated cancer cells and it may be helpful for exploring treatment target of NSCLC.

## Results

**Figure 1:**
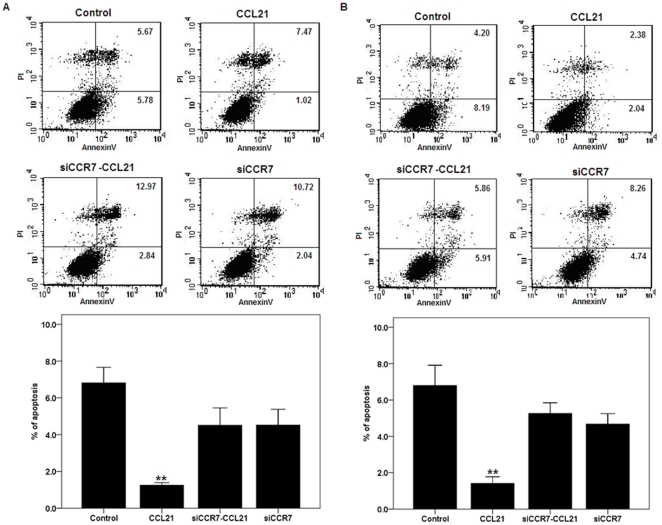
Effect of CCL21/CCR7 on apoptosis in A549 and H460 cells. A549 (A) and H460 (B) cells were treated with CCL21 (100 ng/mL) for 24 h after transfection with CCR7 siRNA (siCCR7). After treatment, apoptosis was estimated using Annexin V staining as described in Methods. Each bar represents the mean ± SD of three independent experiments. **p<0.01, compared with control cells.

CCL21/CCR7 prevents apoptosis of A549 and H460 cells. In our previous study, we identified a higher CCR7 expression level in A549 and H460 human NSCLC cell lines, and its activation promotes cell proliferation [12], [13]. To determine whether activation of CCR7 also influences apoptosis of A549 and H460 cells, CCR7 activation and inhibition were induced with exogenous CCL21 and with CCR7 small interfering RNA (siRNA), respectively [13]. Annexin V staining assay was performed using flow cytometry to determine the effect of CCL21/CCR7 on apoptosis of A549 and H460 cells. As shown in Figure 1, the proportion of pre-apoptotic cells in CCL21 group significantly reduced, compared with the others (all *P*<0.01), while there were no significant differences between the others (all *P*>0.05), implicating that the activation of CCR7 can inhibit apoptosis of A549 and H460 cells whereas the effect of CCL21 can be abolished by the inhibition of CCR7.

**Figure 2:**
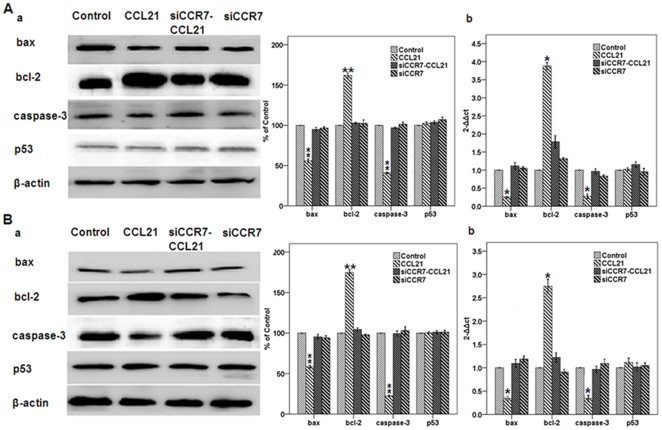
Effect of CCL21/CCR7 on the expression of bcl-2, bax, caspase-3 and p53 in A549 and H460 cells. A549 (A) and H460 (B) cells were treated with CCL21 (100 ng/mL) for 24 h after transfection with CCR7 siRNA (siCCR7). After treatment, the expression of bcl-2, bax, caspase-3 and p53 at both protein (a) and mRNA (b) was estimated using western blot (a) and real-time PCR (b) as described in Methods. Each bar represents the mean ± SD of three independent experiments. *p<0.05 or **p<0.01, compared with control cells.

**Figure 3:**
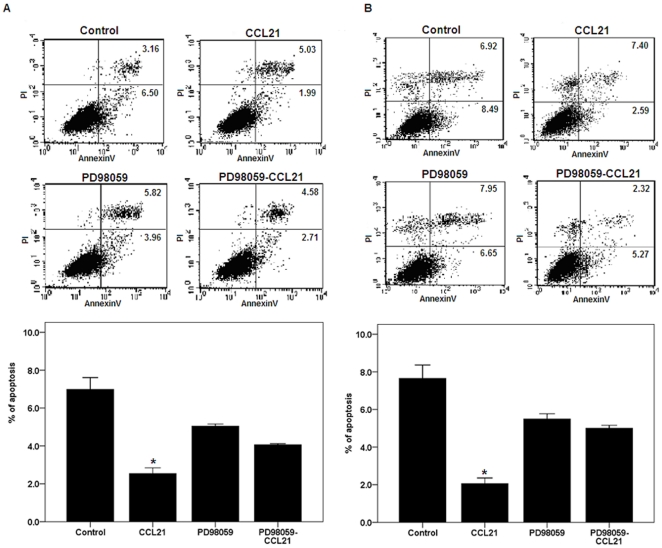
Effect of CCL21/CCR7 on apoptosis of A549 and H460 cells after inhibiting ERK activation. A549 (A) and H460 (B) cells were treated with CCL21 (100 ng/mL) for 24 h after exposure to PD98059, a selective inhibitor of MEK that disrupts activation of downstream ERK, for 1 h. After treatment, apoptosis was estimated using the Annexin V staining. Each bar represents the mean ± SD of three independent experiments. *p<0.05, compared with control cells.

**Figure 4:**
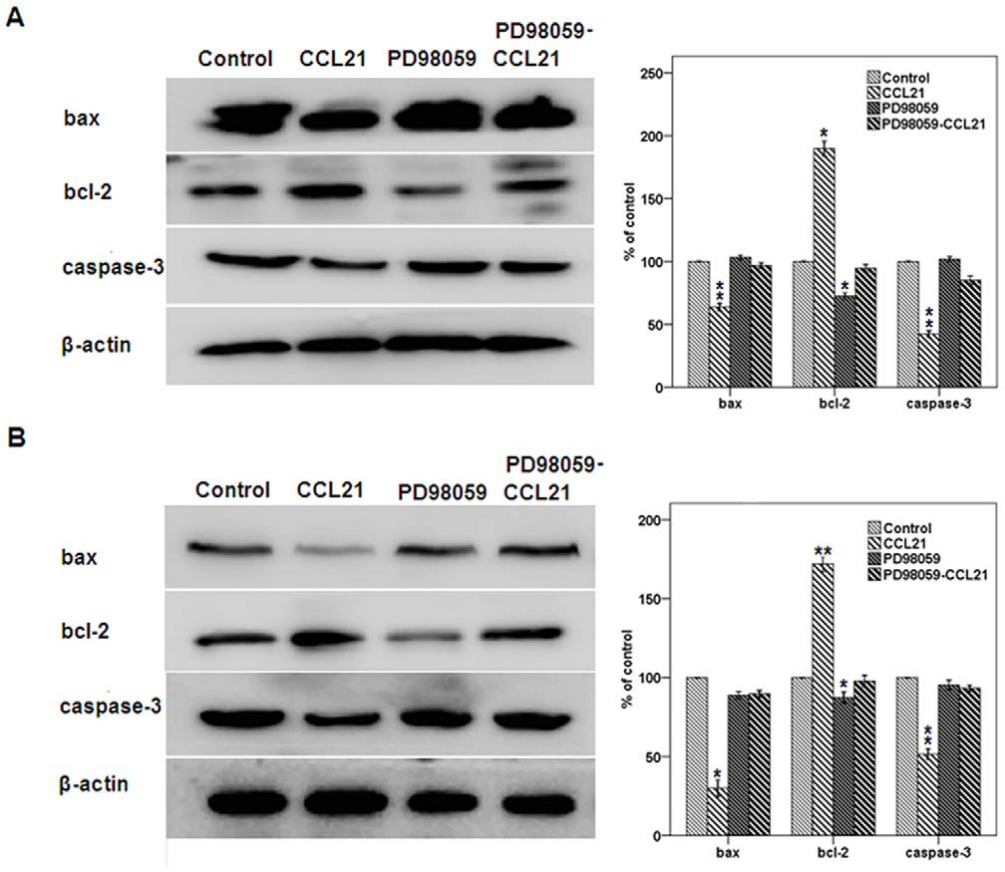
Effect of CCL21/CCR7 on the expression of bcl-2, bax, and caspase-3 after inhibiting ERK activation. A549 (A) and H460 (B) cells were treated with CCL21 (100 ng/mL) for 24 h after exposure to PD98059, a selective inhibitor of MEK that disrupts activation of downstream ERK, for 1 h. After treatment, the expression levels of these components were estimated using western blot. Each bar represents the mean ± SD of three independent experiments. *p<0.05 or **p<0.01, compared with control cells.

**Figure 5:**
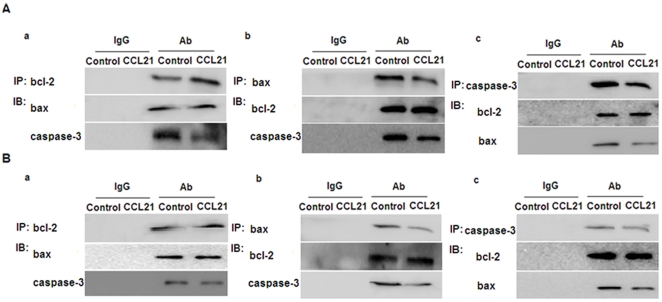
The interaction between bcl-2, bax, and caspase-3 in the absence or presence of CCL21. A549 (A) and H460 (B) cells in the absence or presence of CCL21 (100 ng/mL) for 24 h were subjected to immunoprecipitation with antibodies against bcl-2 or IgG, followed by western blotting for bax and caspase-3 (a). Reciprocal immunoprecipitation with antibodies against bax or IgG were analyzed by western blotting for bcl-2 and caspase-3 (b). Reciprocal immunoprecipitation with antibodies against caspase-3 or IgG were analyzed by western blotting for bcl-2 and bax (c).

Inhibition of apoptosis is involved in regulation of anti-apoptotic bcl-2 and of pro-apoptotic bax and caspase-3 but not p53 in A549 and H460 cells. It has been established that apoptosis within cells is chiefly involved in the expression of bcl-2, bax, caspase-3, or p53 [4],[18]–[20]. To determine possible mechanism by which activation of CCR7 inhibited apoptosis of A549 and H460 cells, western blot and real-time PCR assays were performed. As shown in Figure 2, the expression at both protein and mRNA levels of anti-apoptotic bcl-2 and of pro-apoptotic bax and caspase-3 were respectively upregulated and downregulated in CCL21 group, compared with the others (all *P*<0.01), while there were no significant differences between the others (all *P*>0.05). Interestingly, the expression of pro-apoptotic p53 has not been altered (*P*>0.05). These results indicated that the function of CCR7 as an inhibitor on apoptosis of the NSCLC cells is predominantly implemented possibly by the pathways of bcl-2, bax, and caspase-3 but not p53.

CCL21/CCR7 regulates the expression of bcl-2, bax and caspase-3 via the ERK pathway. Our previous study confirmed that CCL21/CCR7 interaction enhances phosphorylation of ERK (p-ERK) in A549 and H460 cells [13]. To determine whether the ERK pathway is also involved in the anti-apoptotic action of CCL21/CCR7, the cells were treated with PD98059, a selective inhibitor of MEK that disrupts activation of downstream ERK, for 1 h. We found that PD98059 significantly abolished CCL21/CCR7-mediated anti-apoptotic effects (Figure 3) and the alterations in the expression of bcl-2, bax and caspase-3 (Figure 4). Interestingly, PD98059 alone (treatment for 1 h) had a significant effect on the expression of bcl-2 but not on the expression of bax and caspase-3 and the apoptosis. As bax has been shown to be a downstream effector of the bcl-2-regulated pathway of apoptosis and the activation of bcl-2 can promote the release of bax, leading to activation of caspases [21], [22], we sought to examine whether there is an interaction between bcl-2, bax, and capspase-3. Their interactions were confirmed by coimmunoprecipitation results (Figure 5).

**Figure 6:**
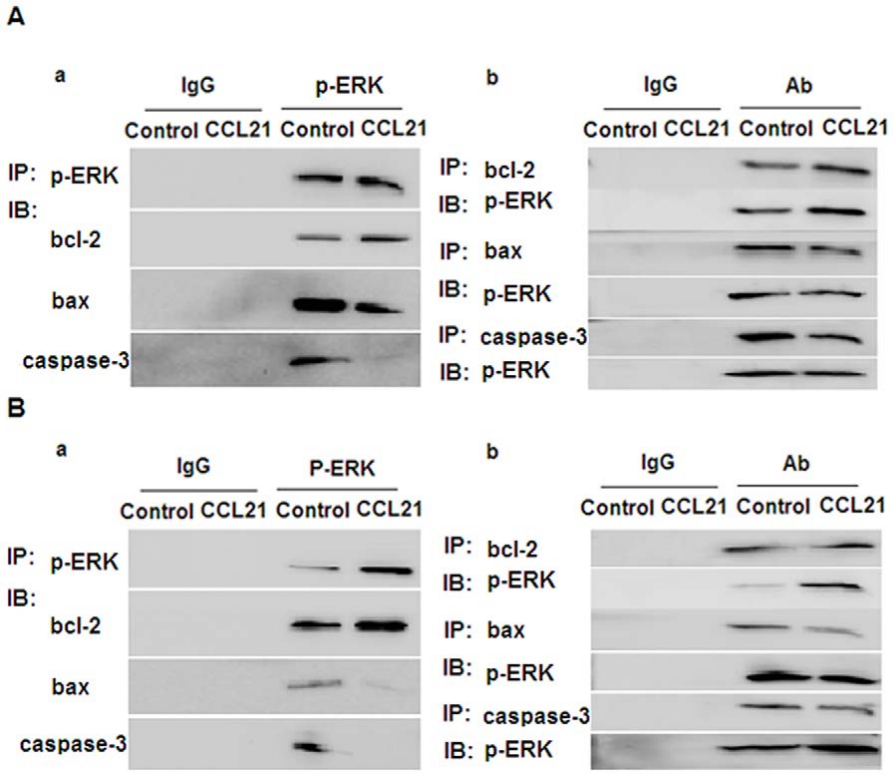
The interaction between p-ERK and bcl-2, bax or caspase-3 in the absence or presence of CCL21. A549 (A) and H460 (B) cells in the absence or presence of CCL21 (100 ng/mL) for 24 h were subjected to immunoprecipitation with antibodies against p-ERK or IgG, followed by western blotting for bcl-2, bax and caspase-3 (a). Reciprocal immunoprecipitation with antibodies against bcl-2, bax, caspase-3 or IgG were analyzed by western blotting for p-ERK (b).

Phosphorylation of ERK, induced by CCL21/CCR7, interacts with bcl-2, bax, or caspase-3. To further identify whether there is an interaction between p-ERK and bcl-2, bax, or caspase-3, coimmunoprecipitation was performed. A549 and H460 cells in the absence or presence of CCL21 for 24 h were subjected to immunoprecipitation with antibodies against p-ERK or IgG, followed by western blotting for bcl-2, bax and caspase-3. A pronounced, specific interaction between p-ERK and bcl-2, bax, or caspase-3 was observed, especially when the cells were treated with CCL21 for 24 h (Figure 6a). Reciprocal immunoprecipitation with antibodies against bcl-2, bax, caspase-3, or IgG was assessed by western blotting for p-ERK, and again, the interaction between p-ERK and bcl-2, bax, or caspase-3 was salient, especially in the presence of CCL21 (Figure 6b).

**Table 1:** Primer sequences used in real-time PCR experiments.

Gene	Primer sequence (5′ – 3′)	Sequence	Product
Bax	F: TTGCTTCAGGGTTTCATCCA	NM_138761	113 bp
	R: AGACACTCGCTCAGCTTCTTG		
Bcl-2	F: TGGCCAGGGTCAGAGTTAAA	NM_000633	147 bp
	R: TGGCCTCTCTTGCGGAGTA		
Caspase-3	F: TGTGGCATTGAGACAGAC	NM_004346	159 bp
	R: CATGGCACAAAGCGACTG		
P53	F: CGTGTGGAGTATTTGGATGACAGA	NM_000546	100 bp
	R: TGTAGTGGATGGTGGTACAGTCAGA		

## Discussion

Our previous studies have documented that activation of CCR7, induced by its ligand CCL21, may promote cell proliferation and mediate its lymph node metastasis in the NSCLC cells [12], [13]. However, the role of CCL21/CCR7 in apoptosis of NSCLC remains vague. In the current study, we demonstrated that CCL21/CCR7 prevents apoptosis in A549 and H460 cells of NSCLC, which is potentially mediated via the ERK pathway.

Bcl-2 and bax are regulators of the cell intrinsic apoptosis pathway, which regulates the integrity of the outer mitochondrial membrane [21], [23], [24]. It has been demonstrated that some bcl-2 family members located on the mitochondrial membrane (such as bax, bcl-XL, Mcl-1, bcl-2, and Bid) can alter the permeability of the mitochondrial membrane and trigger the activation of caspases, leading to apoptotic cell death [25]–[27]. In agreement with results from different cell models [4], [18], [20], [28]–[30], our results showed that activation of CCR7 prevented apoptosis, resulting in an increase in the expression of anti-apoptotic bcl-2 and a decrease in the expression of pro-apoptotic bax and caspase-3. Interestingly, this inhibitory effect was not obviously associated with the pathway of p53. Also, p53 has no significant impact on the expression of CCR7 [31].

Our previous study has indicated that activation of CCR7 can enhance the phosphorylation of ERK in A549 and H460 cells [13], which is consistent with several studies using other cell types [20], [28], [32]–[43]. In this study, we examined the role of phosphorylation of ERK in CCL21/CCR7-mediated anti-apoptosis of A549 and H460 cells. We found that CCL21/CCR7-mediated anti-apoptosis action related to the expression of bcl-2, bax and caspase-3 could be abolished by treatment with PD98059. PD98059 alone (treatment for 1 h) had a significant effect on the expression of bcl-2 but not on the expression of bax and caspase-3 and the apoptosis. Besides, coimmunoprecipitation results confirmed that there was an interaction between bcl-2, bax, and caspase-3. The reason for the absence of alteration in expression of bax and caspase-3 is possibly due to the limitation of observed time because bax and caspase-3 has been shown to be downstream effectors of the bcl-2-regulated pathway of apoptosis [21], [22].

As CCL21/CCR7 was capable of regulating the expression of bcl-2, bax, and caspase-3, we sought to determine whether there is an interaction between p-ERK and bcl-2, bax, or caspase-3. Coimmunoprecipitation and reciprocal immunoprecipitation results strongly suggested that there is an interaction between p-ERK and bcl-2, bax, or caspase-3, especially in the presence of CCL21. These results demonstrate that the effect of CCL21/CCR7 on cell apoptosis involved in the expression of bcl-2, bax and caspase-3 may occur via the ERK pathway in human NSCLC cells.

This study suggests that activation of CCR7, induced by CCL21, can significantly prevent apoptosis of NSCLC cells, which is potentially mediated via the ERK pathway. This information may help clarify the mechanisms of cancer cell survival and identify potential targets for treatment of NSCLC.

## Materials and Methods

### Cell culture and reagents

A549 and H460 cell lines from our previous published paper [12], [13] were cultured in RPMI-1640 or DMEM-F12 supplemented with 10% HyClone fetal bovine serum (FBS) (ThermoFisher Scientific, Fremont, CA, USA) in an atmosphere of 5% CO_2_ at 37°C. Cells were grown in 75 cm^2^ culture flasks and harvested in a solution of trypsin-EDTA at the logarithmic growth phase.

Bcl-2, bax, caspase-3, p-ERK, IgG, and β-actin mouse or rabbit monoclonal antibodies were purchased from Santa Cruz Biotechnology (Santa Cruz, CA, USA). Recombinant human CCL21 was from Pepro Tech (Rocky Hill, NJ, USA). Lipofectamine 2000 was from Invitrogen (Carlsbad, CA, USA). The sequences of siCCR7 and control siRNA and the method of transfection have been reported in another paper [13]. PD98059 was obtained from Sigma (St. Louis, MO, USA) and used at 50 µM (final concentrations) in accordance with previous reports [13], [41], [44], [45]. Protein A/G beads were from Beyotime (Haimen, China).

### Annexin V staining

After treatment with CCL21 for 24 h, cells were harvested and washed twice with cold PBS by gentle shaking. Resuspend cells were added to Binding buffer (1×) and adjusted cell density to 2-5×10^5^/mL. In the dark, 5 µL Annexin V-FITC (50 mM TRIS, 100 mM NaCl, 1% BSA, 0.02% Sodium Azide, pH 7.4) was added to cell suspension Mix of 195 µL and incubated for 10 min at room temperature before adding 190 µL Binding buffer (1×) and 10 µL PI. Ten thousand events per sample were acquired using a FACS-scan flow cytometer (Becton-Dickinson, San Jose, CA, USA) and the percentage of cell apoptosis were analyzed using CellQuest analysis software (Becton-Dickinson).

### Western blot analysis

After treatment with CCL21 for 24 h, cells were extracted with lysis buffer (150 mM NaCl, 1% NP-40, 0.1% SDS, 2 µg/mL aprotinin and 1 mM PMSF) for 30 min at 4°C. Extracts were centrifuged at 12,000×*g* for 15 min at 4°C. Supernatants containing total protein then were harvested. Aliquots, each containing 50 µg proteins, were separated by 12% SDS-PAGE and transferred to PVDF membranes at 40 V for 2 h at low temperature. The membranes were blocked in 5% skim milk for 2 h, and proteins were detected using monoclonal antibodies at 1∶200 dilution overnight at 4°C. Proteins were visualized using anti-mouse or anti-rabbit IgG conjugated with horse radish peroxidase (HRP) at 1∶6000 (caspase-3, p53, bax) or 1∶8000 (the others) dilution for 2 h at room temperature, respectively. Bands were imaged with an EC3 Imaging System (UVP LLC, Upland, CA, USA), and the optical density (OD) was measured using ImageJ (NIH, Bethesda, MD, USA). The OD difference between tested proteins and β-actin of the same sample was calculated as relative content and expressed graphically.

### Real-time PCR

Total RNA was isolated from cells using TRIzol (Invitrogen) according to the manufacturer’s instructions. Real-time PCR was performed on an ABI Prism 7900HT Fast System (Applied Biosystems, Foster, CA, USA) using SYBR Premix Ex Taq II (TaKaRa, Dalian, China). Amplifications were carried out in a total volume of 20 µL and cycled 40 times after initial denaturation (95°C for 30 s) with the following parameters: 95°C for 5 s and 60°C for 30 s. Primers sequences were listed in Table 1 and β-Actin was used as an internal control [13], [46]. The reliability of PCR results was supported by analyzing the dissociation curve. Real-time PCR data were calculated using the 2^-ΔΔCT^ method on the SDS 2.4 software package (Applied Biosystems) [47].

### Coimmunoprecipitation

After treatment with CCL21 for 24 h, cells were extracted with lysis buffer (10 mM KCl, 1.5 mM MgCl_2_, 10 mM HEPES [pH 7.9], 1 mM PMSF, 1 mM DTT) and homogenized for 30 min at 4°C. The extracts were centrifuged at 12,000×*g* for 15 min at 4°C, and the supernatants containing total protein were harvested. Equal amounts of protein were exposed to antibodies against p-ERK, bcl-2, bax and caspase-3 which were immobilized on protein A/G beads. Following 3-h incubation at 4°C with gentle rotation, beads were washed extensively five times with lysis buffer, boiled, and microcentrifuged. Proteins were detected with antibodies against p-ERK, bcl-2, bax and caspase-3 by western blot.

### Statistical analysis

Data were analyzed using SPSS 16.0 software (SPSS Inc., Chicago, IL, USA). A one-way analysis of variance (ANOVA) was used to evaluate the differences between groups with various treatments, and the least significant difference (LSD) test or Dunnett T3 test was used for post hoc subgroup analysis. All data are presented as the mean ± SD of three independent experiments. Results were considered statistically significant for p<0.05. N-fold values for gene expression change up to 0.5 and below 2 were taken as nonsignificant in accordance with values obtained from negative control genes [47], [48].
